# Profiling of Brevetoxin Metabolites Produced by *Karenia brevis* 165 Based on Liquid Chromatography-Mass Spectrometry

**DOI:** 10.3390/toxins13050354

**Published:** 2021-05-14

**Authors:** Huihui Shen, Xiuxian Song, Yue Zhang, Peipei Zhang, Jing Li, Weijia Song, Zhiming Yu

**Affiliations:** 1 CAS Key Laboratory of Marine Ecology and Environmental Sciences, Institute of Oceanology, Chinese Academy of Sciences, Qingdao 266071, China; shenhuihui@qdio.ac.cn (H.S.); yzhang4@qnlm.ac (Y.Z.); zhangpeipei@qdio.ac.cn (P.Z.); lijing@mju.edu.cn (J.L.); songweijia@qdio.ac.cn (W.S.); zyu@qdio.ac.cn (Z.Y.); 2 Laboratory of Marine Ecology and Environmental Science, Qingdao National Laboratory for Marine Science and Technology, Qingdao 266237, China; 3 University of Chinese Academy of Sciences, Beijing 100049, China; 4 Center for Ocean Mega-Science, Chinese Academy of Sciences, Qingdao 266071, China

**Keywords:** *Karenia brevis* 165, profile, brevetoxin metabolites, liquid chromatography-mass spectrometry

## Abstract

In this study, *Karenia brevis* 165 (*K. brevis* 165), a Chinese strain, was used to research brevetoxin (BTX) metabolites. The sample pretreatment method for the enrichment of BTX metabolites in an algal culture medium was improved here. The method for screening and identifying intracellular and extracellular BTX metabolites was established based on liquid chromatography-time-of-flight mass spectrometry (LC-ToF-MS) and liquid chromatography triple quadrupole tandem mass spectrometry (LC-QqQ-MS/MS). The results show that the recovery rates for BTX toxins enriched by a hydrophilic–lipophilic balance (HLB) extraction column were higher than those with a C18 extraction column. This method was used to analyze the profiles of extracellular and intracellular BTX metabolites at different growth stages of *K. brevis* 165. This is the first time a Chinese strain of *K. brevis* has been reported that can produce toxic BTX metabolites. Five and eight kinds of BTX toxin metabolites were detected in the cell and culture media of *K. brevis* 165, respectively. Brevenal, a toxic BTX metabolite antagonist, was found for the first time in the culture media. The toxic BTX metabolites and brevenal in the *K. brevis* 165 cell and culture media were found to be fully proven in terms of the necessity of establishing a method for screening and identifying toxic BTX metabolites. The results found by qualitatively and quantitatively analyzing BTX metabolites produced by *K. brevis* 165 at different growth stages show that the total toxic BTX metabolite contents in single cells ranged between 6.78 and 21.53 pg/cell, and the total toxin concentration in culture media ranged between 10.27 and 449.11 μg/L. There were significant differences in the types and contents of toxic BTX metabolites with varying growth stages. Therefore, when harmful algal blooms occur, the accurate determination of BTX metabolite types and concentrations will be helpful to assess the ecological disaster risk in order to avoid hazards and provide appropriate disaster warnings.

## 1. Introduction

*Karenia brevis* (*K. brevis*), a harmful algal bloom species, can produce potent polyether-type neurotoxins (brevetoxins or BTXs) which impact human health, marine mammals, birds, and fish [1,2,3], and the harmful algal bloom ultimately destroys marine ecosystems and causes economic losses [4,5,6]. BTXs are grouped to A-type compounds including BTX1 and its derivatives and B-type compounds, such as BTX2, BTX3, and other derivatives based on their backbone structure [7,8]. BTX1 and BTX2 are parent toxins in all BTX metabolites. Presently, a variety of BTXs and their derivatives have been discovered in the marine environment. Hydrolyzed and oxidized BTXs have been identified in two strains of *K. brevis* (NOAA and New Pass) and natural blooms [9]. The hydrolyzed BTXs were cytotoxic according to cytotoxic experiments [9]. In addition to BTX metabolites, *K. brevis* also produces other polyether compounds which are nontoxic antagonists that inhibit BTX toxicity, such as tamulamides A and B, brevisin, and brevenal [10,11,12,13]. Therefore, it is necessary to comprehensively identify and quantitatively analyze the profiles of BTX metabolites. This practice is conducive to objectively assessing potential hazards caused by BTX metabolites in order to provide an early warning for ecological disasters.

Establishing an efficient and reliable extraction method is an essential prerequisite for accurately analyzing marine biotoxins in different environments. At present, solid phase adsorption toxin tracking (SPATT) and solid phase extraction (SPE) are commonly used to enrich biotoxins, and SPE is widely used in laboratory research because of its simple operation and short experimental period [14,15,16,17]. The C18 SPE column has been used to enrich and purify liposoluble BTX metabolites with a high content in seawater, *K. brevis* cultures and shellfish samples [9,18,19]. The research by Pan et al. showed that a hydrophilic–lipophilic balance (HLB) column can enrich and clean trace polyether-type marine biotoxins in *P. lima* cultures [20]. Therefore, it is expected to further improve the efficiency of enriching trace BTX metabolites in *K. brevis* cultures based on previous research.

At present, the methods for analyzing enriched BTXs samples include the mouse bioassay (MBA), cytotoxicity assays, the receptor binding assay, enzyme-linked immunosorbent assay (ELISA) and liquid chromatography-mass spectrometry (LC-MS) [21,22,23,24]. LC-MS is widely used because of its high sensitivity and resolution. Liquid chromatography triple quadrupole tandem mass spectrometry (LC-QqQ-MS/MS) offers high sensitivity and is conducive to accurately detecting target compounds with low concentrations in marine environments. Multiple reaction monitoring (MRM) mode and product (PRO) mode can identify compounds according to multi-stage mass spectrometry information. For example, BTX metabolites have been found in *K. brevis* cultures, clams and sediments using the liquid chromatography-tandem mass spectrometry (LC-MS/MS) technique [9,25,26,27]; however, LC-QqQ-MS/MS can judge target compounds and cannot realize the identification of non-targeted toxic metabolites. Liquid chromatography-high resolution mass spectrometry (LC-HR-MS) offers high accuracy and a high resolution, and the technique plays an important role in the discovery and identification of unknown compounds. The LC-HR-MS method has been used to screen and identify targeted and non-targeted lipophilic marine toxins in mussels and *Prorocentrum lima* (*P. lima*) cultures, showing the potential of qualitative analysis with HR-MS [20,28,29]. Liquid chromatography-time-of-flight mass spectrometry (LC-ToF-MS) is a common type of LC-HR-MS. In conclusion, the LC-ToF-MS and LC-QqQ-MS/MS technologies complement each other and can support comprehensive screening and accurate identification of BTX metabolite profiles when considering different environmental samples.

*K. brevis* is widely distributed along the coasts of the world, and the Gulf of Mexico is most seriously damaged by *K. brevis* algal blooms [1,2,30,31,32]. In different sea areas of the world, the BTX metabolites produced by diverse *K. brevis* strains vary in terms of the types and contents [33]. In China, harmful *K. brevis* algal blooms have broken out in the coastal areas of Zhejiang Province, and this alga has been detected in the coastal areas of Guangdong Province and the Yangtze Estuary [34,35]; however, BTX metabolites have not been reported offshore from China or as being produced by Chinese algae strains. Furthermore, the types of BTX metabolites produced by Chinese algae strains and the differences in their ability to produce BTX metabolites at growing phases are not clear. The toxins metabolites produced by different toxigenic algae at growth stages are also different, resulting in varying degrees of environmental risk [36]. At present, the algae strain *K. brevis* 165 has been identified in Daya Bay in China. The BTX metabolites may cause ecological environment hazards, as seen when considering that *K. brevis* has bloomed in China. Here, the solid-phase extraction method for enriching BTX metabolites in culture media is optimized based on LC-MS technology. The established method is used to characterize and quantify BTX metabolites produced by *K. brevis* 165 from the exponential phase to the decline phase. Ultimately, this research could complement scientific research on the toxic strains of *K. brevis*, and can be helpful to assess the ecological disaster risk in order to avoid hazards and provide appropriate disaster warnings.

## 2. Results and Discussion

### 2.1. Optimized Detection and Enrichment Conditions of BTXs

#### 2.1.1. Detection Method of LC-MS

In an acidic system, LC-MS/MS methods can simultaneously separate and detect different types of BTXs [18,37], but the sensitivity and precision of LC-ToF-MS for BTX metabolites have not been completely reported. The detection precision for BTXs by LC-ToF-MS is shown in Table S1. For BTX1, BTX2, and BTX3, the relative standard deviations (RSDs) of the peak area were ≤7.00%, the RSDs of the retention time were ≤0.47%, and the mass deviation of measured exact mass were within 3.74 ppm. Those results prove that LC-ToF-MS has good precision and can be used to identify BTX metabolites. The precision of LC-QqQ-MS/MS showed that the instrument meets the detection requirements (Table S2). The results for the matrix standard curve method that were used to quantitatively detect BTXs in *K. brevis* cells and culture media by LC-QqQ-MS/MS are shown in Tables S3-1 and S3-2, respectively. According to the results above, all of the correlation coefficients (R²) of the BTXs were ≥0.999, indicating that BTXs exhibited good linearity in their quantitative linear ranges. In conclusion, LC-QqQ-MS/MS met the requirement of accurate quantitative analysis of BTX metabolites in *K. brevis* cells and culture media.

#### 2.1.2. Enrichment Method for BTXs in *K. brevis* Culture Media

**Figure 1:**
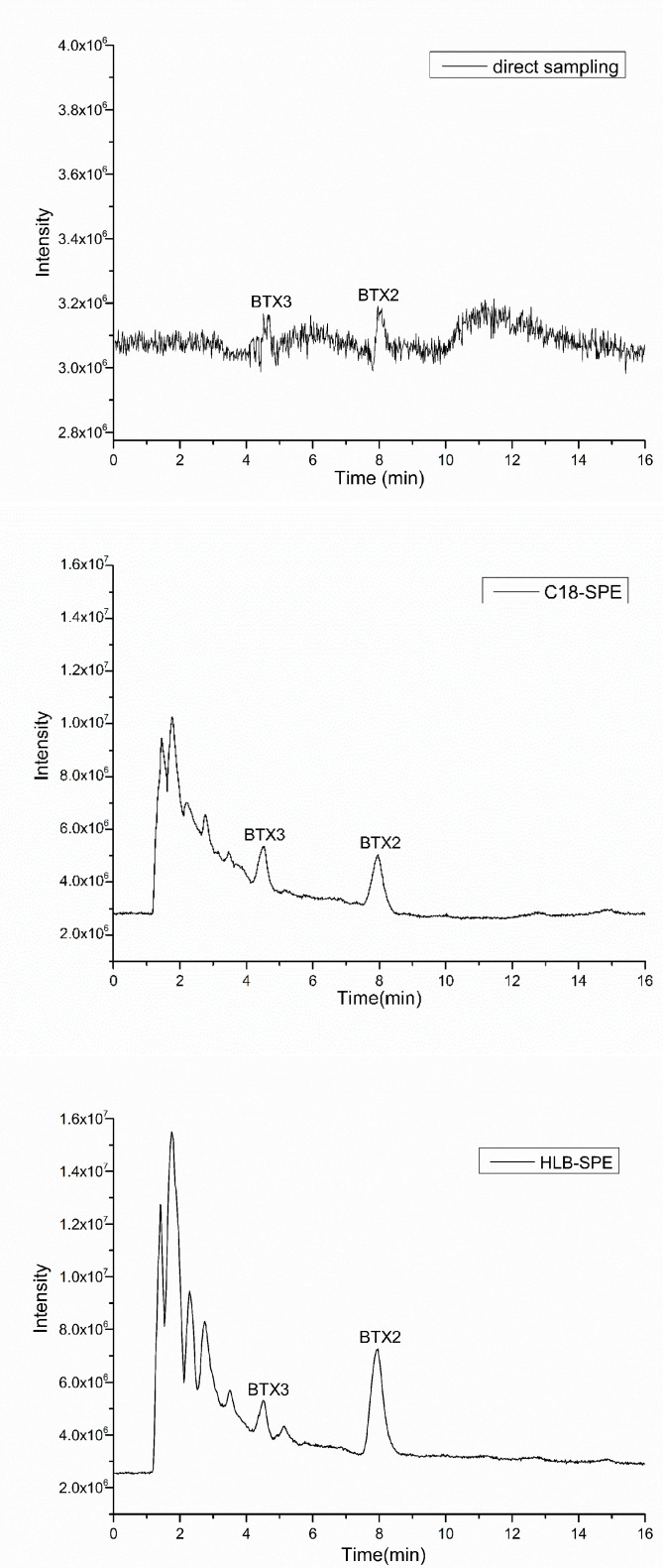
TICs (*m/z* 200–1300) with the LC-ToF-MS method for *K. brevis* culture media treated by direct sampling, C18 and HLB solid-phase extraction.

In this study, the pretreatment methods in which HLB and C18 extraction columns were used to enrich BTXs in *K. brevis* culture media were optimized and compared based on the previous enrichment methods of lipophilic algae toxins [20]. The total ion chromatograms (TICs) with the LC-ToF-MS method for the same *K. brevis* culture media were enriched by the HLB and C18 extraction columns, as shown in Figure 1. Compared with the direct sampling method, the peak intensity and signal-to-noise ratio (*SNR*) of BTX2, BTX3, and low-concentration compounds in *K. brevis* culture media were significantly increased after solid-phase extraction (HLB and C18 extraction columns). The extracted ion chromatograms (EICs) of BTX2 with [M+H]^+^ (*m/z* 895.42–895.52) showed that the peak shapes of BTX2 after solid-phase extraction were good (Figure S1). Therefore, HLB and C18 solid-phase extraction could obviously remove impurities in the culture media and improve the sensitivity for detecting target compounds. In conclusion, HLB and C18 solid-phase extraction to enrich BTX metabolites is helpful for finding new compounds in culture media. 

**Figure 2:**
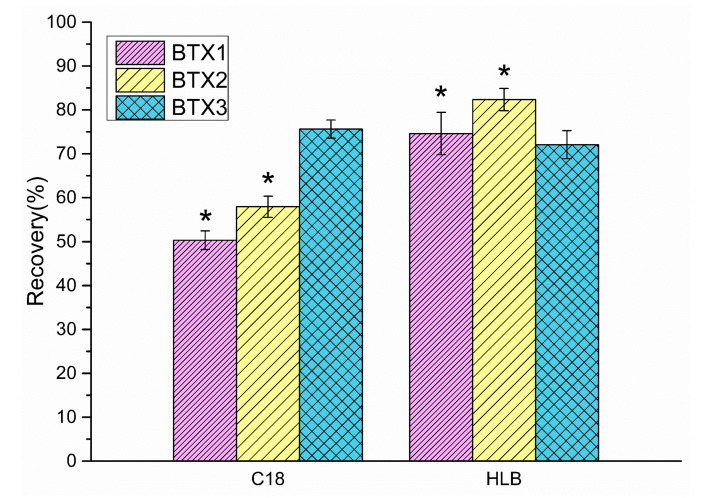
Recovery of mixed standard solution BTXs diluted with *Karenia mikimotoi* (*K. mikimotoi)* culture media as treated by C18 and HLB extraction columns. Error bar is the result of triple analysis. * denotes significant difference between C18 and HLB extraction columns when BTX1 and BTX2 in culture media were enriched (*p* < 0.05).

Additionally, the recovery and stability with solid-phase extraction to enrich BTX1, BTX2, and BTX3 in culture media were studied. The results are shown in Figure 2. The recoveries of BTX1, BTX2, and BTX3 in culture media enriched by the C18 extraction column were 50.31%, 57.95%, and 75.64%, respectively, and the average RSDs (n = 3) were ≤2.44%. The recoveries of BTX1, BTX2, and BTX3 in culture media enriched by HLB extraction column were 74.61%, 82.36%, and 72.08%, respectively, with average RSDs (n = 3) ≤4.82%. Furthermore, we used SPSS 22.0 to analyze the significant difference between C18 and HLB solid-phase extraction. The analysis results show that C18 and HLB solid-phase extraction were significantly different when BTX1 and BTX2 in culture media were enriched (*p* < 0.05), while the difference was not significant when BTX3 was enriched (*p* > 0.05). The above results show that compared with C18 solid-phase extraction, the recovery of BTX1 and BTX2 when enriched by HLB solid-phase extraction significantly increased, while the recovery of BTX3 was almost the same. A possible reason for this is that the divinylbenzene structure in the HLB extraction column packs more easily when combined with BTX1 and BTX2 rather than when using the C18 method. Considering that BTX1 and BTX2 are both parent toxins and that the content of BTX2 is high in algal cells and cultures, the HLB extraction column was selected to enrich and purify BTX metabolites in *K. brevis* culture media.

### 2.2. Comprehensive Screening and Identification of Intracellular and Extracellular BTX Metabolites of K. brevis

**Figure 3:**
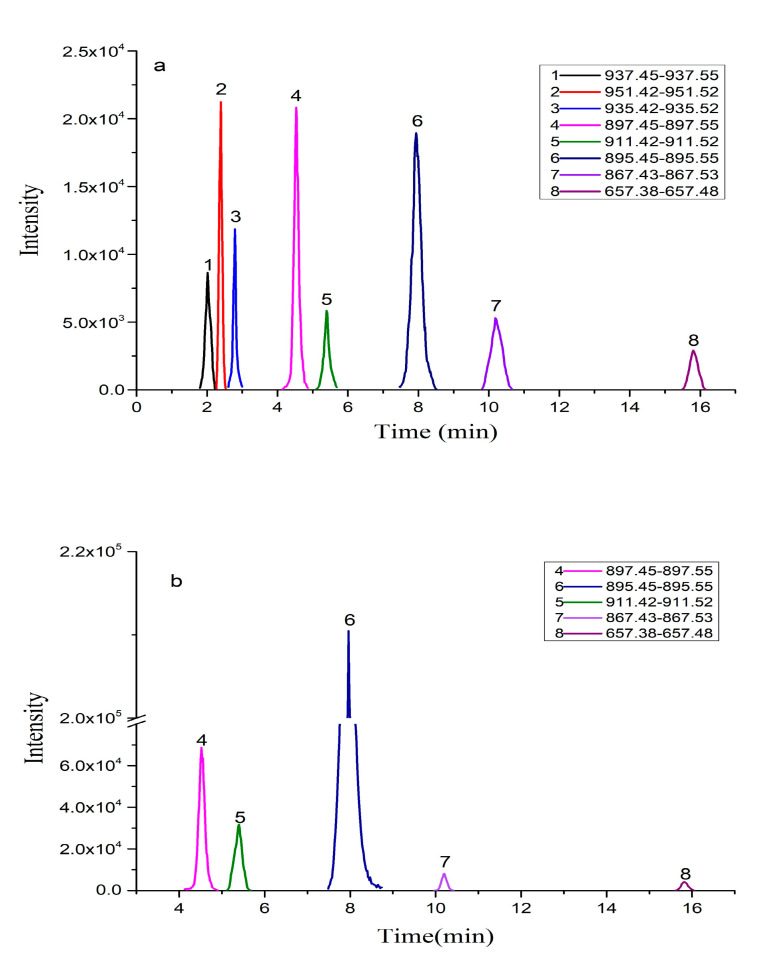
EICs of suspected BTX metabolites in samples with LC-ToF-MS method in the positive ion mode. (**a**) *K. brevis* culture media treated by HLB extraction column; (**b**) *K. brevis* algal cells extracted by methanol.

The pretreated *K. brevis* 165 culture samples and algal cell samples were fully scanned by LC-ToF-MS in the positive ion mode. According to the accurate relative molecular weights of [M+H]^+^, [M+NH_4_]^+^, [M+Na]^+^, and [M+K]^+^ shown in Table S4, the EICs of the suspected BTX metabolites in samples were obtained as shown in Figure 3.

#### 2.2.1. Identification of BTX1, BTX2, and BTX3 with Reference Standards

**Figure 4:**
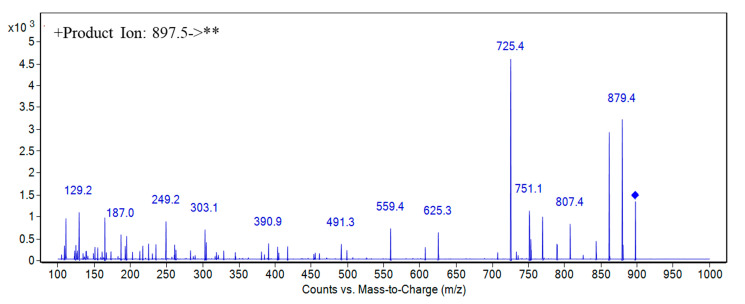
MS/MS spectra of the peak 4 compound (the precursor [M+H]^+^ ion: *m/z* 897.5) in Figure 3 with LC-QqQ-MS/MS method. ** denotes fragment ions of precursor [M+H]^+^ ion (*m/z* 897.5).

For BTX1, BTX2, and BTX3, the retention time, first-order mass spectrometric information, and multi-stage mass spectrometric information of suspected compounds in *K. brevis* 165 culture media and algae cell samples were compared with the reference standards. For example, the retention time of 4.5 min, exact mass of 897.5028, and the multi-stage fragment information in the second-order mass spectrometry of peak 4 in Figure 3 were all consistent with BTX3 (Figure 4), so it was judged that the peak 4 compound is BTX3. In the same way, it can be deduced that the peak 6 compound is BTX2 and peak 7 is BTX1. Therefore, Figure 3 shows that BTX1, BTX2, and BTX3 were found in the *K. brevis* 165 algal cells and culture media. The study by Errera et al. demonstrated that BTX1, BTX2, and BTX3 may be detected in the algae cells and culture media of six strains of *K. brevis* (Wilson, TXB3, TXB4, SP1, SP2, SP3, NSP3, NBK) [38]; however, Lekan et al. found that BTX1 and BTX2 produced by *K. brevis* Wilson were only found in cells, while BTX3 was only detected in culture media [39]. The work by Waggett et al. showed that *K. brevis* SP1 does not produce quantifiable BTX metabolites [40]. It can be concluded that BTX1, BTX2, and BTX3 as produced by *K. brevis* are ubiquitous in most strains, but different culture conditions may lead to differences in the types, distributions, and contents of BTXs produced by different strains [40].

#### 2.2.2. Identification of BTX Metabolites without Reference Standards

**Figure 5:**
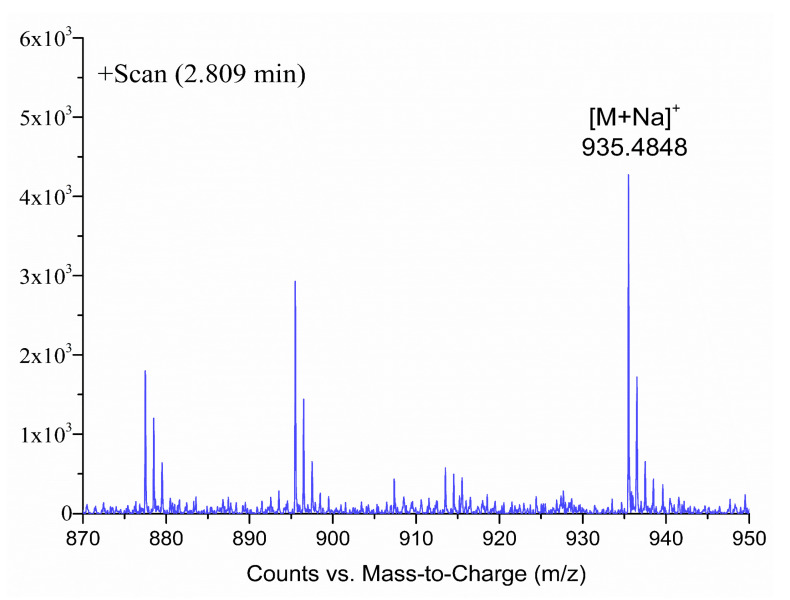
LC-ToF-MS spectra information for the peak 3 compound in Figure 3.

**Figure 6:**
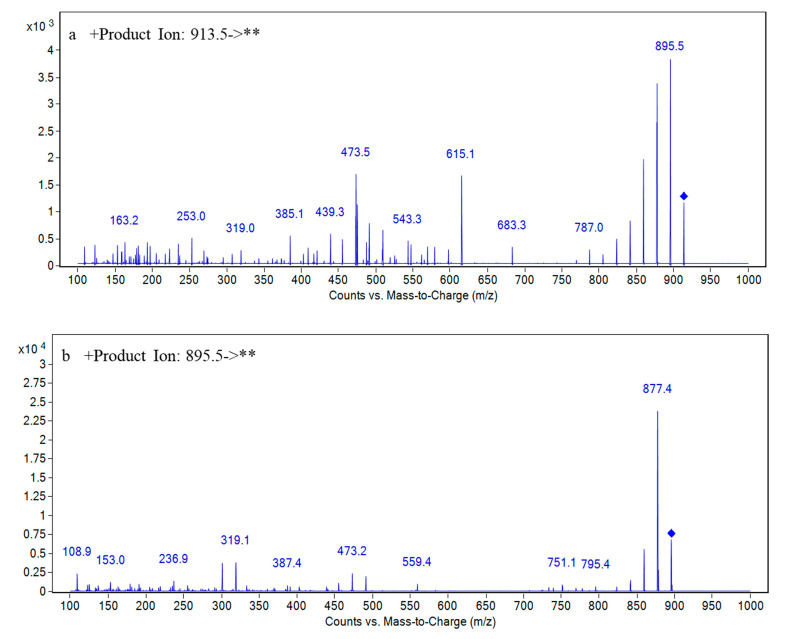
MS/MS spectra information for the peak 3 compound in Figure 3 (the precursor [M+H]^+^ ion: *m/z* 913.5) (**a**) and BTX2 (the precursor [M+H]^+^ ion: *m/z* 895.5) (**b**). ** denotes fragment ions of precursor [M+H]^+^ ion.

For the BTX metabolites without reference standards, peak 3 in Figure 3 was taken as an example to illustrate the process of screening and identifying metabolites in detail. The accurate mass weight of the peak 3 compound in Figure 3 was *m/z* 935.4848 (Figure 5) based on the EIC by the extraction window *m/z* 935.42–935.52 of [M+Na]^+^. The molecular formula for peak 3 was inferred by Masshunter as C_50_H_72_O_15_, which was the same as open-ring BTX-2 with one more H_2_O than BTX2; meanwhile, the relative mass error was ≤ 10 ppm. It was preliminarily determined that the peak 3 compound may be open-ring BTX-2. In summary, LC-ToF-MS technology can be used to screen and preliminarily identify BTX metabolites in cells and culture media of *K. brevis*. The LC-QqQ-MS/MS method was used to further verify the suspected open-ring BTX-2 (peak 3 compound) in Figure 3. Figure 6a shows the MS/MS spectrum of the suspected open-ring BTX-2 ([M+H]^+^). A typical fragment ion (*m/z* 895) of peak 3 compound was consistent with the quasi-molecular ion ([M+H]^+^) of BTX2 as compared to the MS/MS spectrum of BTX2 (Figure 6b), and peak 3 compound and BTX2 had same fragment ion (*m/z* 473). In addition, the other fragment ions at peak 3 were 18 larger than the corresponding fragment ions of BTX2, and 18 represents H_2_O here. The result shows that the peak 3 compound was the hydrolysate of BTX2. Therefore, the peak 3 compound was ultimately confirmed as open-ring BTX-2. The MS/MS spectra information for open-ring BTX-2 in the *K. brevis* NOAA and New Pass algal cells and *K. brevis* bloom water is consistent with this paper [9]; however, BTX metabolites without standard substances that have only been identified by LC-MS/MS may produce inaccurate results. Therefore, the combination of LC-ToF-MS and LC-QqQ-MS/MS can accurately identify BTX metabolites without a reference standard and when considering unknown compounds.

The identification results and MS/MS spectra of BTX metabolites in *K. brevis* 165 algal cells and culture media are shown in Table 1 and Figure S2, respectively. Four kinds of BTX metabolites, including BTX2, BTX3, BTX1, BTX-B5, and brevenal, an antagonist of BTX toxins [13], were found in *K. brevis* 165 algal cells and have been identified in other *K. brevis* strains [33,39]. Among BTX metabolites, BTX2, which can convert into other metabolites, is the most widely distributed metabolite in natural environments and is the parent toxin of the BTX2 family. Seven BTX metabolites (BTX2, BTX3, BTX1, BTX-B5, OR-BTX2, OR-BTX-B5, and OR-BTX3) and an antagonist named brevenal were found in the *K. brevis* 165 culture media. It is noteworthy that brevenal was first discovered in *K. brevis* culture media. Lekan et al. found that brevenal only exists in the algal cells of *K. brevis* Wilson [39]. The brevenal distribution varies between *K. brevis* 165 and *K. Brevis* Wilson, which may be due to different toxigenic characteristics among different strains. The presence of brevenal in culture media may counteract the toxicity of BTXs and reduce the environmental risk caused by BTX metabolites in culture media.

### 2.3. BTX Metabolites Contents Produced by K. brevis at Different Growth Stages

Here, the types and contents of extracellular and intracellular BTX metabolites produced by *K. brevis* 165 at different growth stages were determined by LC-MS, and the results are shown in Table S5. The single-cell toxin contents (intracellular and extracellular toxins) and BTX metabolite concentrations in culture media showed an increasing trend with an increase in culture time. At different growth stages of *K. brevis* 165, BTX2 had the highest single-cell toxin concentration (intracellular and extracellular toxins) with a range from 3.54 to 15.64 pg/cell. The BTX2 content of *K. brevis* Wilson has been found to be more than 12 pg/cell, which is similar to the results of this study [33]. In addition, brevenal was found for the first time in *K. brevis* culture media, and its concentration was less than 1.05 μg/L at different growth stages. The concentration of brevenal in single cells and culture media increased as culture time was prolonged. In conclusion, the content of BTX metabolites and brevenal changes with the growth of *K. brevis* 165.

**Figure 7:**
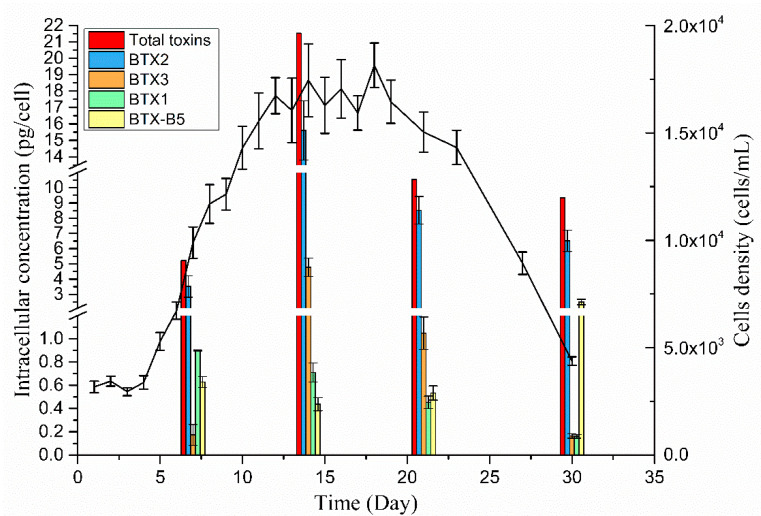
The intracellular BTX metabolite concentrations of *K. brevis* 165 at different times throughout growth (the exponential growth stage on the 7th day, the platform stage on the 14th day, the early decline phase on the 21st day and the end of decline phase on the 30th day). Error bar is the result of triple analysis.

At different growth stages of *K. brevis* 165, the changes in intracellular BTX metabolites are shown in Figure 7, illustrating that the total single-cell content of intracellular toxins in *K. brevis* 165 increased first and then decreased and finally stabilized. The total intracellular toxin single-cell content at the exponential growth stage of *K. brevis* 165 was 6.78 pg/cell, and it was lowest at the algal growth stage. At the exponential growth stage of *K. brevis* 165, the nutrients in the culture media are abundant. As the algae cells divide rapidly, the toxins are continuously synthesized; however, the number of cells increases rapidly, so the total single-cell content of intracellular toxins reaches a minimum. At the platform stage, the *K. brevis* 165 algal cell division rate slowed down, the cell density was almost unchanged, and the accumulation rate of intracellular toxins tended to halt. Therefore, the total intracellular toxin content reached a maximum with 21.53 pg/cell. During the decline phase of *K. brevis* 165, the algae apoptosis rate was higher than the generation rate, and the apoptosis promotes a passive release of BTX metabolites. At this stage, a large number of algal cells rupture and intracellular BTX metabolites are released into the culture, leading to the total extracellular BTX metabolite content continually increasing. Therefore, the total content of intracellular BTX toxin metabolites rose from the exponential phase to the stable phase and then decreased in the decline phase of *K. brevis* 165.

In addition, *K. brevis* 165’s total toxin content (intracellular and extracellular toxins) increased, indicating that BTX metabolites were continuously produced and that the toxin generation rate was greater than the decomposition rate during the culture process. Table S6 shows that the proportion of intracellular BTX metabolites produced by *K. brevis* 165 varied between growth stages. The proportion of BTX2 in algal cells varied from 51.95% to 83.13%. BTX2 was a major BTX metabolite in *K. brevis* 165 cells, which is consistent with the study of Pierce et al. [41]. From the exponential growth phase to the platform phase, the proportion of intracellular BTX2 in total intracellular toxins increased, while the proportion of BTX2 decreased during the decline period. BTX2 is the parent toxin of B-type brevetoxins, so the toxin is synthesized first and has the highest content in cells. During the decline period, the lack of nutrients in the culture media slows down the generation rate of the mother toxin, while the content of BTX2 derivatives increases. Therefore, the proportion of intracellular BTX2 decreased and the proportion of BTX-B5 increased during the decline period. Similarly, the proportion of intracellular BTX1 in the decay period was lower than that of other growth stages.

**Figure 8:**
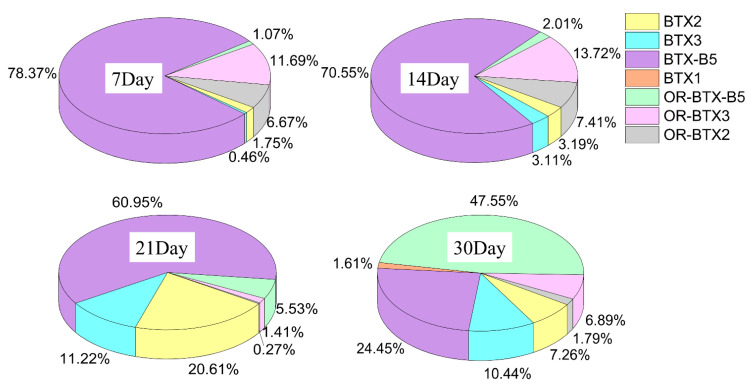
The proportion of extracellular BTX metabolites produced by *K. brevis* 165 at different times throughout growth (the exponential growth stage on the 7th day, the platform stage on the 14th day, the early decline phase on the 21st day and the end of decline phase on the 30th day).

Both the release of intracellular BTX metabolites and the transformation of extracellular BTX metabolites resulted in the changing of BTX metabolite types in the culture media. Figure 8 shows that the proportion of extracellular BTX metabolites produced by *K. brevis* 165 varied between growth stages. The concentration of BTX-B5 in *K. brevis* 165 culture media was highest from the exponential growth period to the early decline phase. The proportion of BTX-B5 between total BTX metabolites in cultures varied from 78.37% to 60.95%, indicating that the proportion of the toxin decreased with the increase in the culture time. The proportion of OR-BTX-B5 in the cultures ranged from 1.07% to 47.05%, showing an increasing trend in the process of cultivation. In the decline phase, the contents of OR-BTX-B5 in the culture media were higher than those of BTX-B5. A possible reason for this is that the intracellular BTX-B5 released into the culture increased, resulting in an increase in the concentration of OR-BTX-B5 in the culture, even exceeding the content of BTX-B5. Even when the extracellular BTX2 content is low, the toxicity of BTX2 derivatives cannot be ignored [9]. Therefore, it is very important to analyze BTX derivatives in culture media.

The total toxin concentrations of toxic extracellular BTX metabolites throughout *K. brevis* 165 growth varied from 10.27 to 449.11 μg/L is shown in Table S5. In the platform and decline periods, the contents of BTX2 in cultures were 47.70 and 32.62 μg/L, respectively. Pierce et al. [41] showed that the highest content of BTX2 was 42 μg/L in Siesta Beach seawater during *K. brevis* harmful algal blooms, which was similar to the results of this study. When *K. brevis* harmful algal blooms occur, the algal density is generally 1.65 × 10^5^–1.25 × 10^7^ cells/L [41]. Here, the cultured *K. brevis* 165 algal density was in the range of 4 × 10^6^–1.8 × 10^7^ cells/L, which is equivalent to the typical algal density in the field. Therefore, it was inferred that when harmful *K. brevis* 165 algal blooms occur, the concentration of BTX metabolites in seawater may reach tens to hundreds of μg/L, which poses a great threat to various marine organisms, especially cultured shellfish. The hazards of BTX metabolization in seawater cannot be ignored.

## 3. Conclusions

In this study, a modified method based on the combination of LC-ToF-MS and LC-QqQ-MS/MS was established to analyze the intracellular and extracellular BTX metabolites produced by *K. brevis*. The established method provides a methodological basis for further studying environmental behaviors and comprehensively assessing toxicity risk for BTX metabolites. This method was used to rapidly identify and further determine the BTX metabolites produced by the Chinese strain of *K. brevis* 165 at different growth stages as cultured in the laboratory. The results show that five kinds of intracellular BTX metabolites and eight kinds of extracellular BTX metabolites were identified, of which brevenal was found for the first time in a culture media. The total BTX metabolite contents of single cell (intracellular and extracellular toxins) showed an increasing trend, while the intracellular BTX metabolite contents increased at the beginning and then decreased with the increase in the *K. brevis* 165 culture time. In the platform phase, the contents of intracellular BTX metabolites reached a maximum value of 21.53 pg/cell. The concentrations of BTX metabolites in culture media were highest in the decline phase, reaching 449.11 μg/L. These results prove that the BTX metabolites produced by *K. brevis* 165 vary at different growth stages, and the metabolite concentrations cannot be ignored. Therefore, the toxins produced by toxicogenic algae in offshore waters should be accurately monitored to reduce the harm caused by harmful algal blooms and prevent the occurrence of marine ecological disasters.

## 4. Experimental Materials and Methods

### 4.1. Culture of Karenia Brevis

*K. brevis* 165, provided by Xiamen University, was isolated from Daya Bay in Shenzhen and cultured in the algae species bank of the key Laboratory of Marine Ecology and Environmental Science, Institute of Oceanography, Chinese Academy of Sciences. The *K. brevis* 165 samples in the exponential growth period were inoculated in seawater with a salinity of about 30‰ and an added f/2 media [42]. The initial cellular concentrations of *K. brevis* 165 were about 5000 cells L^–1^. The cultures were cultivated under a light intensity of 40–50 μmol photons/ (m^2^·s) and a light–dark cycle of 12:12 h with a temperature of 21 ± 1 °C.

### 4.2. Sample Preparation 

#### 4.2.1. Extraction of Intracellular BTX Metabolites

Volumes of *K. brevis* 165 algae and culture media were separated through a Whatman GF/D filter membrane (47 mm, GE Healthcare, Gaithersburg, MD, USA), and the filter membrane was added into a E lysing matrix tube (4.5 mL, MP Biomedicals, Solon, OH, USA) with 3.0 mL of methanol. The algal cells were fragmented by environmental sample function of automated nucleic acid extraction instrument (MP Biomedicals, Solon, OH, USA) and then the mixture was ultrasonically extracted for 30 min at 20 ± 2 °C, followed by centrifugation at 8000 rpm for 10 min. The supernatant was transferred to another 15 mL centrifuge tube. The algal cell precipitates were extracted again according to the previously mentioned procedure and the extracts were combined. The combined sample was dried under a nitrogen stream and then reconstituted with 1 mL of methanol. The methanol was filtered through one nylon membranes with a poor size of 0.22 μm prior to determination.

#### 4.2.2. Extraction of BTX Metabolites in Cultures by Solid-Phase Extraction

The *K. brevis* culture media were loaded onto Waters HLB (3 cc, 60 mg) and Waters C18 (200 mg, 3 mL) solid-phase extraction columns with a flow rate of 1 mL/min. The extraction columns were preconditioned with 6.0 mL of methanol and 6.0 mL of methanol/water (20:80, *V*/*V*). Subsequently, the extraction columns were rinsed with 3.0 mL of methanol/water (10:90, *V*/*V*) to remove undesirable compounds, and 6.0 mL of methanol containing a 0.3% formic acid solution was used to elute the target BTX metabolites. The collected eluents were dried under a nitrogen stream and then redissolved with 1 mL of methanol. The methanol was filtered through 0.22 μm nylon membranes prior to determination.

### 4.3. LC-MS Conditions

#### 4.3.1. LC-ToF-MS Conditions

The BTX metabolites were separated by a 1200 Series LC system (Agilent Technologies, Wilmington, DE, USA) with a ZORBAX Extend-C18 column (150 mm × 3.0 mm, 3.5 μm), quaternion pump, diode array detector, automatic sample injector, etc. The gradient elution was processed at room temperature (20 ± 2 °C), with ultrapure water as mobile phase A and acetonitrile as mobile phase B. Both mobile phases A and B contained 0.1% formic acid. The flow rate was 0.4 mL min^–1^ and the injection volume was 5 μL. The binary gradient elution was performed as follows: The initial concentration was 20% B, then 30% B at 15 min, 47.5% B at 20 min, 58% B at 25 min, and 20% B at 30 min. The stop time was 30 min and the post time was 5 min.

The BTX metabolites were screened and preliminarily identified in positive ion modes by a G1969A ToF-MS (Agilent Technologies, Wilmington, DE, USA) equipped with an ESI source. The mass spectrometer conditions were applied as follows: scan range of 200–1300 *m/z*; nebulizer pressure (N_2_) of 40 psi; drying gas (N_2_) temperature and flow rate of 350 °C and 11 L/min, respectively; capillary voltage of 4.5 KV. The voltage condition for the mass spectrometer, including the fragmentor was 120 V and the skimmer voltage was 60 V.

#### 4.3.2. LC-QqQ-MS/MS Conditions

The BTX metabolites were separated by an Agilent 1290 Infinity Ⅱ Series LC system (Agilent Technologies, Wilmington, DE, USA) with an Eclipse Plus C18 column (50 mm × 2.1 mm, 3.5 μm), G7120A binary pump, G7167B automatic sample injector, G7117B diode array detector, etc. The gradient elution was processed at room temperature (20 ± 2 °C), with ultrapure water as mobile phase A and acetonitrile as mobile phase B. Both mobile phases A and B contained 0.1% formic acid. The flow rate was 0.3 mL/min and the injection volume was 10 μL. The binary gradient elution was performed as follows: The initial concentration was 50% B, then 72% B at 12 min, 100% B at 16 min, 50% B at 18 min, and 20% B at 30 min. The stop time was 18 min, and the post time was 3 min.

The BTX metabolites were analyzed in terms of their structures and concentrations by a 6470 QqQ-MS/MS (Agilent Technologies, Wilmington, DE, USA) equipped with an ESI source in the product (PRO) mode. The mass spectrometer conditions were consistent with the nebulizer pressure (N_2_), capillary voltage and drying gas (N_2_) temperature of ToF-MS conditions, and the gas flow rate was 7 L min^–1^.

### 4.4. Data Analysis

#### 4.4.1. Identification of BTX Metabolites

The mass spectrum data for BTX metabolites have been summarized in the literature, including molecular formulae for compounds and accurate molecular weights of ion peaks produced in both positive and negative mode of mass spectrometry, including the [M+H]^+^, [M+NH^4^]^+^, [M+Na]^+^, [M+K]^+^ and [M−H]^−^ data. The relevant details are shown in Table S4. The TICs data for intracellular and extracellular samples of *K. brevis* were obtained by full scan analysis in the positive mode with the LC-ToF-MS method. The Masshunter software package (A02.02) was used to obtain the EICs (*SNR* > 5) according to the information for compounds listed in Table S4. For BTX1, BTX2, and BTX3, the names of the suspected compounds were determined by comparing the retention times (the peak time of the suspected compound and the standard compound changed within ± 0.20 min) and the multi-stage fragment information of the suspected compound with the standard compound. For other suspected BTX metabolites, Masshunter (A02.02) was used to deduce the molecular formula (the relative standard deviation of mass was set within 10 ppm). Then, the suspected compounds were initially identified through nitrogen rules and comprehensive scores. Finally, the relationships of suspected compounds and BTX1, BTX2, and BTX3 were determined by comparing the fragment data for the initially identified compounds to the standards based on the LC-QqQ-MS/MS method.

#### 4.4.2. Quantitative and Semi-Quantitative Methods for BTX Metabolite Analysis

Under the PRO mode with a QqQ-MS/MS, the matrix standard curve with the external standard method was used for quantitative and semi-quantitative analysis of BTX metabolites in *K. brevis* algal cells and culture samples [10]. The retention times, precursor ions, and qualitative and quantitative ions of BTX1, BTX2, and BTX3 are shown in Table S7.

## Figures and Tables

**Table 1:** The identification results of BTX metabolites in *K. brevis* 165 algal cells and culture media.

Peak	Retention Time (min)	Toxins	Molecular Formula	Detected Ion	Observed Mass (*m/z*)	Theoretial Mass (*m/z*)	Mass Error (ppm)	Score	Toxins Distribution
1	2.0	Open-ring BTX3	C_50_H_74_O_15_	[M+Na]^+^	937.4991	937.4920	−7.77	98.43	Extracellular
2	2.4	Open-ring BTX-B5	C_50_H_72_O_16_	[M+Na]^+^	951.4792	951.4713	−8.55	98.1	Extracellular
3	2.8	Open-ring BTX2	C_50_H_72_O_15_	[M+Na]^+^	935.4848	935.4763	−9.27	97.74	Extracellular
4	4.5	BTX3	C_50_H_72_O_14_	[M+H]^+^	897.5028	897.4995	−3.7	99.85	Intracellular/extracellular
5	5.4	BTX-B5	C_50_H_70_O_15_	[M+H]^+^	911.4856	911.4787	−7.53	98.52	Intracellular/extracellular
6	8.0	BTX2	C_50_H_70_O_14_	[M+H]^+^	895.4867	895.4838	−3.2	100	Intracellular/extracellular
7	10.2	BTX1	C_49_H_70_O_13_	[M+H]^+^	867.4907	867.4889	−2.06	100	Intracellular/extracellular
8	15.8	Brevenal	C_39_H_60_O_8_	[M+H]^+^	657.4426	657.4361	−9.91	97.74	Intracellular/extracellular

## Data Availability

Data are available upon request; please contact the contributing authors.

## Supplementary Materials

The following are available online at https://www.mdpi.com/article/10.3390/toxins13050354/s1, Figure S1: The LC-ToF-MS EICs of BTX2 with [M+H]^+^ (*m/z* 895.42–895.52) in *K. brevis* 165 culture media treated by two methods. Figure S2. MS/MS spectra of BTX metabolites in *K. brevis* 165 algal cells and culture media with LC-QqQ-MS/MS method. Table S1: The experimental results of ToF-MS instrument precision. Table S2: The experimental results of MS/MS instrument precision. Table S3-1. The inspection results of detecting BTXs in *K. brevis* 165 cells by LC-QqQ-MS/MS. Table S3-2. The inspection results of detecting BTXs in *K. brevis* 165 culture media by LC-QqQ-MS/MS. Table S4: Formula and theoretical precise molecular mass of 34 BTX metabolites. Table S5: The changes in concentrations of BTX metabolites produced by *K. brevis* 165 at different times throughout growth. Table S6: The proportion of intracellular BTX metabolites produced by *K. brevis* 165 at different times throughout growth. Table S7: Parameters of MS/MS in PRO mode for OA and DTX1 toxins.
