# Supplementary material for: Profiling of Brevetoxin Metabolites Produced by Karenia brevis 165 Based on Liquid Chromatography-Mass Spectrometry
Source: Toxins (Basel). 2021 May 14;13(5):354. doi: 10.3390/toxins13050354 (PMC8156667; doi:10.3390/toxins13050354)
Supplement: Supplementary file 1 [file toxins-13-00354-s001.zip › toxins-1190063-supplementary.pdf]

Supplementary Materials: Profiling of Brevetoxin Metabolites Produced by *Karenia brevis* 165 Based on Liquid Chromatography-Mass Spectrometry

Huihui Shen, Xiuxian Song, Yue Zhang, Peipei Zhang, Jing Li, Weijia Song and Zhiming Yu

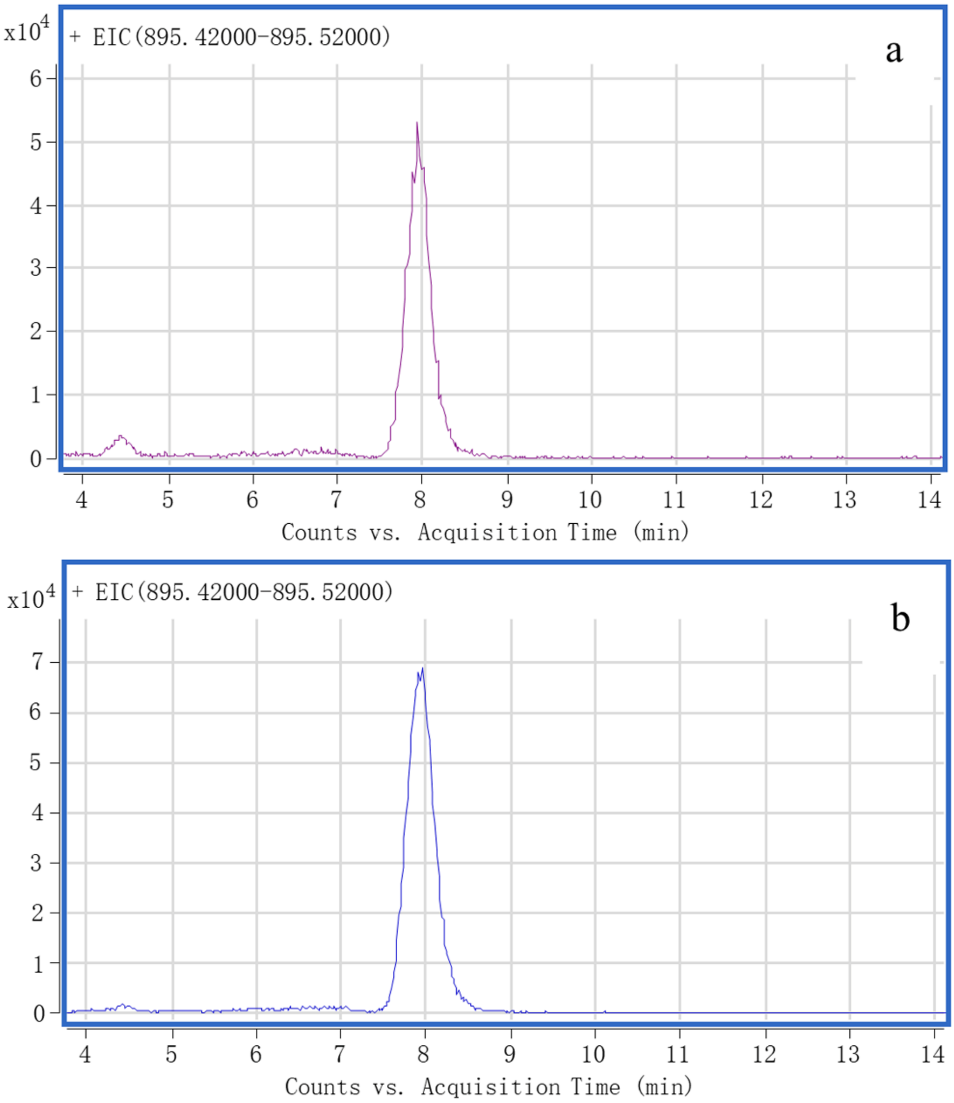

Figure S1. The LC-ToF-MS EICs of BTX2 with $[M+H]^+$ (*m/z* 895.42–895.52) in *K. brevis* 165 culture media treated by two methods. (**a**) C18 solid-phase extraction; (**b**) HLB solid-phase extraction.

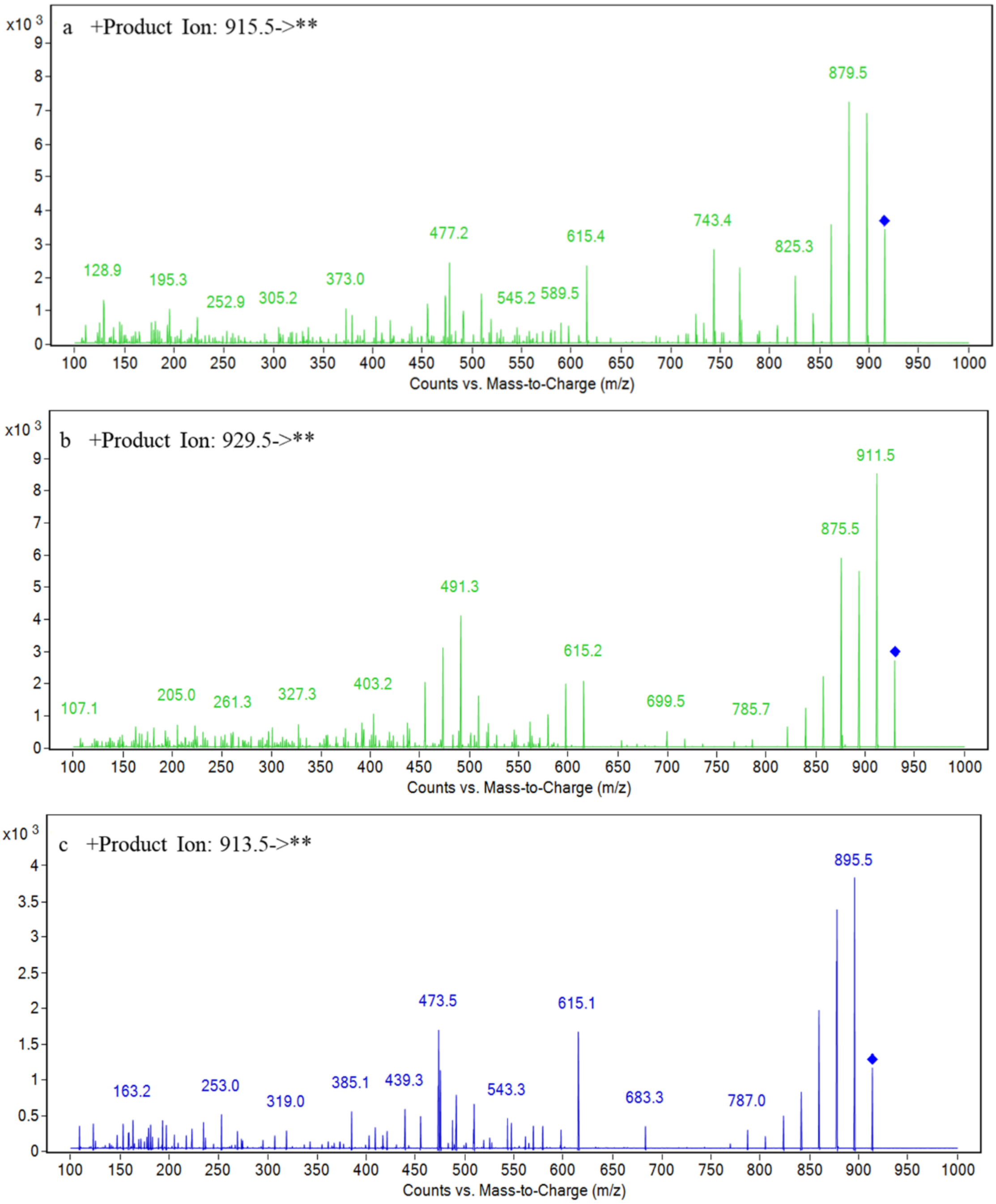
a +Product Ion: 915.5->**
x10 3
128.9
195.3
252.9
305.2
373.0
477.2
545.2
589.5
615.4
743.4
825.3
879.5
Counts vs. Mass-to-Charge (m/z)
b +Product Ion: 929.5->**
x10 3
107.1
205.0
261.3
327.3
403.2
491.3
615.2
699.5
785.7
875.5
911.5
Counts vs. Mass-to-Charge (m/z)
c +Product Ion: 913.5->**
x10 3
163.2
253.0
319.0
385.1
439.3
473.5
543.3
615.1
683.3
787.0
895.5
Counts vs. Mass-to-Charge (m/z)

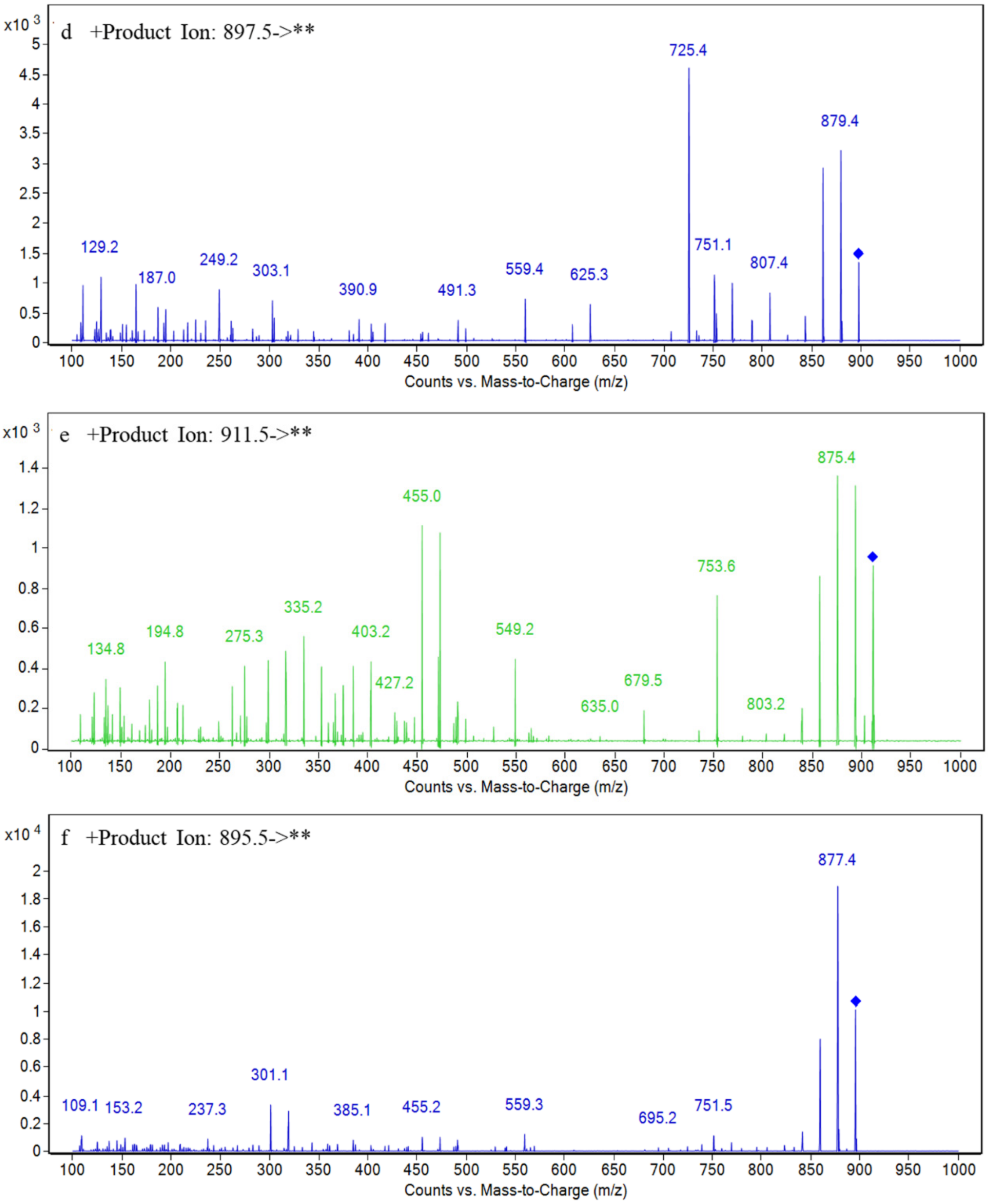
x10 3
d +Product Ion: 897.5->**
725.4
879.4
129.2
187.0
249.2
303.1
390.9
491.3
559.4
625.3
751.1
807.4
Counts vs. Mass-to-Charge (m/z)
x10 3
e +Product Ion: 911.5->**
875.4
455.0
753.6
335.2
134.8
194.8
275.3
403.2
427.2
549.2
635.0
679.5
803.2
Counts vs. Mass-to-Charge (m/z)
x10 4
f +Product Ion: 895.5->**
877.4
301.1
109.1
153.2
237.3
385.1
455.2
559.3
695.2
751.5
Counts vs. Mass-to-Charge (m/z)

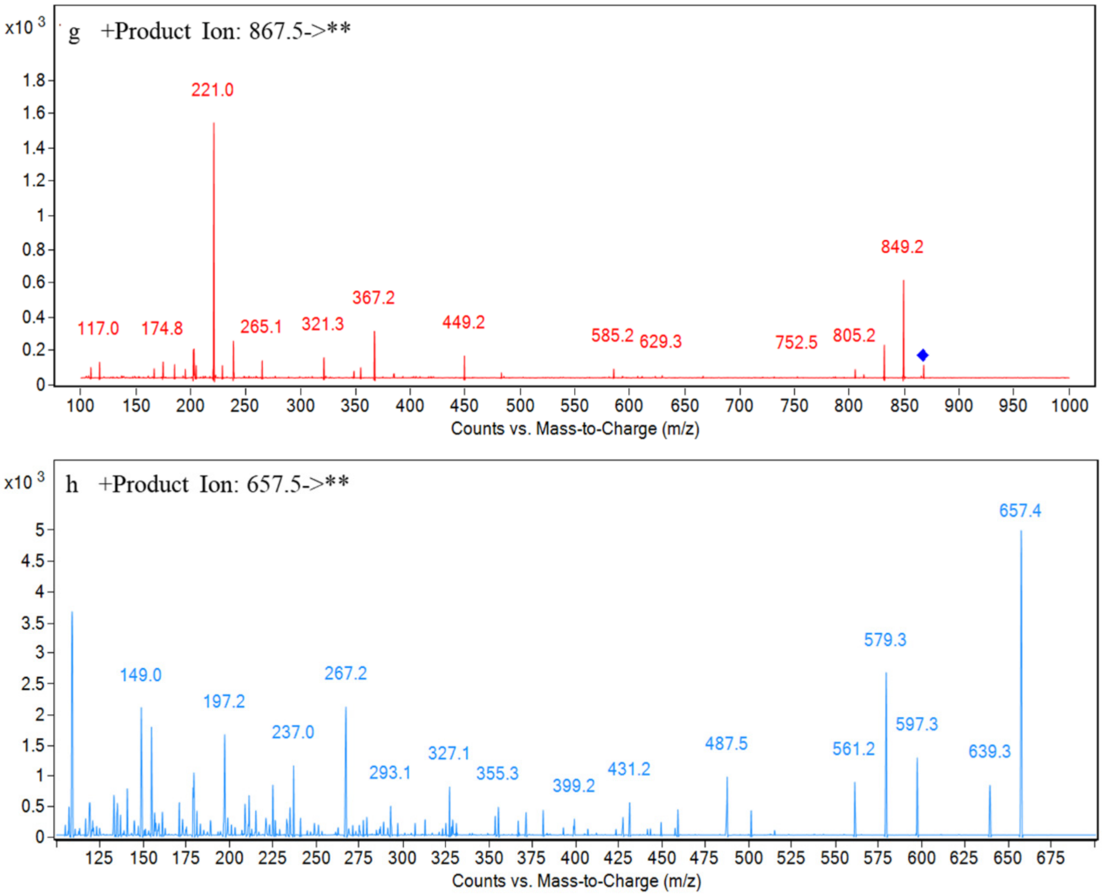

Figure S2. MS/MS spectra of BTX metabolites in *K. brevis* 165 algal cells and culture media with the LC-QqQ-MS/MS method. (**a**: OR-BTX3; **b**: OR-BTX-B5; **c**: OR-BTX2; **d**: BTX3; **e**: BTX-B5; **f**: BTX2; **g**: BTX1; **h**: Brevenal). ** denotes fragment ions of precursor $[M+H]^+$ ion.

Table S1. The experimental results of the ToF-MS instrument precision.

Toxins		1	2	3	4	5	6	RSD (%)
BTX1	Area	47811	46301	43619	39827	45921	48437	7.00
	Retention time (min)	10.191	10.149	10.107	10.163	10.114	10.138	0.31
	Mass error（ppm）	−3.12	−3.31	−3.46	−3.02	−3.59	−3.05	-
BTX2	Area	21057	21952	23715	19974	22763	20163	6.85
	Retention time (min)	7.994	7.962	8.003	7.907	7.929	7.969	0.47
	Mass error（ppm）	−3.18	−3.13	−3.38	−3.27	−3.64	−2.98	-
BTX3	Area	11756	13883	13064	11937	12734	12862	6.13
	Retention time (min)	4.518	4.523	4.507	4.54	4.498	4.531	0.34
	Mass error（ppm）	−3.22	−3.31	−3.74	−3.28	−3.43	−3.09	-

Table S2. The experimental results of the MS/MS instrument precision.

Toxins		1	2	3	4	5	6	RSD (%)
BTX1	Area	12533	12203	12050	11964	12221	12995	3.09
	Retention time (min)	9.341	9.319	9.315	9.302	9.306	9.336	0.17
BTX2	Area	37781	37919	37118	37486	36364	39050	2.38
	Retention time (min)	7.937	7.911	7.898	7.885	7.894	7.928	0.26
BTX3	Area	14483	14564	13836	13847	13985	14714	2.76
	Retention time (min)	4.978	4.957	4.939	4.931	4.939	4.978	0.42

Table 3. The inspection results of detecting BTXs in *K. brevis* 165 cells by LC-QqQ-MS/MS.

BTXs	Linear range (pg)	Calibration curve	Correlation coefficients (R^2)	LOD (pg)	LOQ (pg)	ME (%)
BTX1	75–5000	y = 2.618x − 55.727	0.9999	30	75	+9.32
BTX2	50–5000	y = 7.9439x + 4.4827	0.9999	25	50	+5.71
BTX3	50–5000	y = 4.2403x − 15.772	0.9998	25	50	+6.04

Table S3-2. The inspection results of detecting BTXs in *K. brevis* 165 culture media by LC-QqQ-MS/MS.

BTXs	Linear range (pg)	Calibration curve	Correlation coefficients (R^2)	LOD (pg)	LOQ (pg)	ME (%)
BTX1	100–5000	y = 38.686x − 627.39	0.9999	50	100	+11.62
BTX2	75–5000	y = 87.76x − 6917	0.9992	50	75	+9.83
BTX3	75–5000	y = 72.595x − 7007.5	0.9996	50	75	+10.15

Table S4. Formula and theoretical precise molecular mass of 34 BTX metabolites.

BTX metabolites	Formula	Molecular weights	[M+H]+	[M+NH4]+	[M+Na]+	[M+K]+	[M−H]-
PbTX-1	$C_{49}H_{70}O_{13}$	866.4816	867.4889	884.5155	889.4709	905.4448	865.4744
PbTX-2	$C_{50}H_{70}O_{14}$	894.4766	895.4838	912.5104	917.4658	933.4397	893.4693
PbTX-3	$C_{50}H_{72}O_{14}$	896.4922	897.4995	914.5260	919.4814	935.4554	895.4849
PbTX-5	$C_{52}H_{74}O_{15}$	938.5028	939.5101	956.5366	961.4920	977.4659	937.4955
PbTX-6	$C_{52}H_{72}O_{16}$	952.4820	953.4893	970.5159	975.4713	991.4452	951.4748
PbTX-7	$C_{49}H_{71}O_{13}$	867.4895	868.4967	885.5233	890.4787	906.4526	866.4822
PbTX-8	$C_{49}H_{69}ClO_{14}$	916.4376	917.4450	934.4714	939.4268	955.4007	915.4303
PbTX-9	$C_{50}H_{74}O_{14}$	898.5079	899.5151	916.5417	921.4971	937.4710	897.5006
PbTX-10	$C_{49}H_{74}O_{13}$	870.5130	871.5202	888.5468	893.5022	909.4761	869.5057
PbTX-11	$C_{52}H_{72}O_{14}$	920.4922	921.4995	938.5260	943.4814	959.4554	919.4849
PbTX-12	$C_{55}H_{70}O_{14}$	954.4766	955.4838	972.5104	977.4658	993.4397	953.4693
PbTX-13	$C_{55}H_{78}O_{16}$	994.5290	995.5363	1012.5628	1017.5182	1033.4921	993.5217
PbTX-14	$C_{54}H_{76}O_{15}$	964.5179	965.5257	982.5523	987.5076	1003.4816	963.5112
PbTX-tbm	$C_{46}H_{66}O_{13}$	826.4503	827.4576	844.4842	849.4396	865.4135	825.4431
Brevenal	$C_{39}H_{60}O_{8}$	656.42882	657.4361	674.46264	679.4180	695.3920	655.4215
brevenal acetal	$C_{41}H_{66}O_{9}$	702.4707	703.4780	720.5045	725.4599	741.4338	701.4634
Open-ring PbTx-1	$C_{49}H_{72}O_{14}$	884.4922	885.4995	902.5260	907.4814	923.4554	883.4849
Oxidized PbTx-1	$C_{49}H_{70}O_{14}$	882.4766	883.4838	900.5104	905.4658	921.4397	881.4693
Open-ring, ox. PbTx-1	$C_{49}H_{72}O_{15}$	900.4871	901.4944	918.5210	923.4763	939.4503	899.4799
Open-ring PbTx-7	$C_{49}H_{73}O_{14}$	885.5000	886.5073	903.5339	908.4893	924.4632	884.4928
Open-ring PbTx-2	$C_{50}H_{72}O_{15}$	912.4871	913.4944	930.5210	935.4763	951.4503	911.4799
BTX-B5	$C_{50}H_{70}O_{15}$	910.4715	911.4788	928.5053	933.4607	949.4346	909.4642
Open-ring, BTX-B5	$C_{50}H_{72}O_{16}$	928.4820	929.4893	946.5159	951.4713	967.4452	927.4748
Open-ring PbTx-3	$C_{50}H_{74}O_{15}$	914.5028	915.5101	932.5366	937.4920	953.4659	913.4955
Open-ring cysteine-PbTx-A	$C_{52}H_{81}NO_{16}S$	1007.5276	1008.5349	1025.5614	1030.5168	1046.4908	1006.5203
Open-ring cysteine-PbTx-B	$C_{53}H_{81}NO_{17}S$	1035.5225	1036.5298	1053.5564	1058.5117	1074.4857	1034.51524
BTX-B1	$C_{52}H_{74}NO_{17}SNa$	1039.4575	1040.4648	1057.4913	1062.4467	1078.4207	1038.4502
BTX-B2	$C_{53}H_{80}NO_{17}S$	1034.5147	1035.5220	1052.5485	1057.5039	1073.4779	1033.5074
BTX-B3	$C_{64}H_{96}O_{17}$	1136.6648	1137.6720	1154.6986	1159.6540	1175.6279	1135.6575
N-acylated-BTXB2	$C_{69}H_{110}NO_{18}S$	1272.7444	1273.7516	1290.7782	1295.7336	1311.7075	1271.7371

N-myristoyl-BTXB2	$C_{67}H_{106}NO_{18}S$	1244.7131	1245.7203	1262.7469	1267.7023	1283.6762	1243.7058
cysteine-PbTx	$C_{53}H_{80}NO_{16}S$	1018.5198	1019.5271	1036.5536	1041.5090	1057.4829	1017.5125
cysteine-PbTx sulfoxide	$C_{53}H_{80}NO_{17}S$	1034.5147	1035.5220	1052.5485	1057.5039	1073.4779	1033.5074
taurine metabolite of PbTx-2	$C_{52}H_{76}NO_{17}S$	1018.4834	1019.4907	1036.5172	1041.4726	1057.4466	1017.4761

Table S5. The changes in concentrations of BTX metabolites produced by *K. brevis* 165 at different times throughout growth.

**Incubation Time (day)**		**7**	**14**	**21**	**30**
Algae density ($\times 10^3$ cells/mL)		9.89	17.45	15.04	4.37
BTX2 (pg/cell)	Intracellular	3.52	15.60	12.39	6.52
	Extracellular	1.82×10^{-2}	3.37×10^{-2}	2.90	7.47
	Total	3.54	15.64	15.29	13.98
	Intracellular/Extracellular	193.74	462.87	4.27	0.87
Extracellular concentrations (μg/L)		0.18	0.43	47.70	32.62
BTX3 (pg/cell)	Intracellular	1.73	4.78	1.53	0.16
	Extracellular	0.47×10^{-2}	3.29×10^{-2}	1.58	10.73
	Total	1.74	4.81	3.11	10.89
	Intracellular/Extracellular	365.98	145.20	0.97	1.53×10^{-2}
Extracellular concentrations (μg/L)		4.68×10^{-2}	0.42	25.98	46.88
BTX1 (pg/cell)	Intracellular	0.90	0.71	0.45	0.16
	Extracellular	-	-	-	1.66
	Total	0.90	0.71	0.45	1.82
	Intracellular/Extracellular	-	-	-	9.7×10^{-2}
Extracellular concentrations (μg/L)		4.68×10^{-2}	0.42	25.98	46.88
BTX-B5 (pg/cell)	Intracellular	0.63	0.44	0.53	2.50
	Extracellular	0.81	0.75	8.58	25.12
	Total	1.44	1.18	9.11	27.63
	Intracellular/Extracellular	0.77	0.59	6.22×10^{-2}	0.10
Extracellular concentrations (μg/L)		8.05	9.49	141.07	109.79
Brevenal (pg/cell)	Intracellular	0.42	0.28	0.18	0.62
	Extracellular	2.35×10^{-2}	3.07×10^{-2}	5.53×10^{-2}	0.24
	Total	0.44	0.31	0.23	0.86
	Intracellular/Extracellular	17.79	8.97	3.22	2.58
Extracellular concentrations (μg/L)		0.23	0.39	0.91	1.05
OR-BTX2 (pg/cell)	Extracellular	6.92×10^{-2}	7.83×10^{-2}	3.80×10^{-2}	1.84
	Total	6.92×10^{-2}	7.83×10^{-2}	3.80×10^{-2}	1.84
Extracellular concentrations (μg/L)		6.85×10^{-2}	9.97×10^{-2}	6.25×10^{-2}	8.06
OR-BTX-B5 (pg/cell)	Extracellular	1.11×10^{-2}	2.13×10^{-2}	0.78	48.87
	Total	1.11×10^{-2}	2.13×10^{-2}	0.78	48.87
Extracellular concentrations (μg/L)		0.11	0.27	12.80	213.55
OR-BTX3 (pg/cell)	Extracellular	0.12	0.15	0.20	7.08
	Total	0.12	0.15	0.20	7.08

Extracellular concentrations (μg/L)	1.20	1.85	3.26	30.95
Intracellular total concentrations (pg/cell)	6.78	21.53	14.91	9.35
Extracellular total concentrations (μg/L)	10.27	13.46	231.44	449.11
Total concentrations in single cell (pg/cell)	7.82	22.58	28.98	112.12
Total concentrations (ng/ml)	77.34	394.02	435.86	489.96

Table S6. The proportion of intracellular BTX metabolites produced by *K. brevis* 165 at different times throughout growth.

	**7 day**	**14 day**	**21 day**	**30 day**
BTX2	51.95%	72.49%	83.13%	69.74%
BTX3	25.51%	22.18%	10.25%	1.75%
BTX-B5	9.26%	2.03%	3.58%	26.78%
BTX1	13.27%	3.30%	3.04%	1.72%

Table S7. Parameters of MS/MS in PRO mode for OA and DTX1 toxins.

**Compound**	**Molecular formula**	**Retention time （min）**	**Precursor ions （m/z）**	**Qualitative and quantitative ions （m/z）**	**Fragmentor**
BTX1	$C_{49}H_{70}O_{13}$	10.2	$[M+H]^+$ 867.5	849.7 / 221.0	180
BTX2	$C_{50}H_{70}O_{14}$	8.0	$[M+H]^+$ 895.5	877.5 / 859.5	180
BTX3	$C_{50}H_{72}O_{14}$	4.5	$[M+H]^+$ 897.5	879.5 / 725.5	180

1. Evaluation of Matrix Effect (ME)

The Fujian strain of *Karenia mikimotoi* (*K. mikimotoi*) cultured in the laboratory belongs to *Karenia* spp., and we have proved that the algae do not produce BTX metabolites. So, *K. mikimotoi* cells and culture media treated by methanol and solid-phase extraction, respectively, and the two kinds of extracts were selected as the blank control to evaluate matrix effect of determining BTX metabolites produce by *K. brevis* 165. A mixed standard of BTX1, BTX2 and BTX3 was diluted to the same concentration with methanol and *K. mikimotoi* extracts. Then, the BTX1, BTX2 and BTX3 in three standard solutions were analyzed under the same LC-QqQ-MS/MS conditions. The ME was calculated according to the following formula:

$$ME\ (\%) = \frac{(Ax - As)}{As} \times 100 \quad (1)$$

In the formula, Ax represents the peak areas of the BTXs in the blank algae culture and blank algae cells and As represents the peak areas of the BTXs in methanol. ME below 0% indicates signal suppression, while that above 0% reveals signal enhancement [1, 2].

2. Analyzing Data of Quantitatively Detecting BTX Metabolites

The mixed standard solution of BTX1, BTX2 and BTX3 was diluted with *K. mikimotoi* extracts at different multiples, and the BTX metabolites were detected under the optimum LC-QqQ-MS/MS conditions. Then, BTX metabolites concentration in the *K. brevis* 165 cells and culture media was analyzed based on the method of Shen et al. [1].

3. Recovery Rates

The mixed standard solution of BTX1, BTX2 and BTX3 was diluted with *K. mikimotoi* culture

media to three concentration including 1 μg/L, 10 μg/L and 100 μg/L. Then, the culture media added standards was treated by solid phase extraction (SPE). Each concentration was analyzed in three parallel tests. The concentration of BTX1, BTX2 and BTX3 were detected by LC-QqQ-MS/MS to calculate the recovery rate of the SPE method.

References

1. Shen, H.H.; Chen, J.H.; Xu, X.L., Pan, L.; Wang, X.R. Development of a High Performance Liquid Chromatography-Tandem Mass Spectrometry Method for Determination of Lipophilic Toxins in Marine Shellfishes and Edible Safety Evaluation. *Chin. J. Anal. Chem.* **2018**, *46*, 985–992; doi:10.1016/S1872-2040(18)61092-8.
2. Pan, L.; Chen, J.H.; Shen, H.H.; He, X.P.; Li, G.J.; Song. X.C.; Zhou. D.S.; Sun, C.J. Profiling of Extracellular Toxins Associated with Diarrhetic Shellfish Poison in *Prorocentrum lima* Culture Medium by High-Performance Liquid Chromatography Coupled with Mass Spectrometry. *Toxins* **2017**, *9*, 308:1–308:18; doi:10.3390/toxins9100308.
